# Cell-Based Reporter System for High-Throughput Screening of MicroRNA Pathway Inhibitors and Its Limitations

**DOI:** 10.3389/fgene.2018.00045

**Published:** 2018-02-27

**Authors:** Katerina Brustikova, David Sedlak, Jana Kubikova, Ctibor Skuta, Katerina Solcova, Radek Malik, Petr Bartunek, Petr Svoboda

**Affiliations:** ^1^Institute of Molecular Genetics of the Czech Academy of Sciences, Prague, Czechia; ^2^CZ-OPENSCREEN, Institute of Molecular Genetics of the Czech Academy of Sciences, Prague, Czechia

**Keywords:** miRNA, high-throughput screening, let-7, miR-30, Argonaute

## Abstract

MicroRNAs (miRNAs) are small RNAs repressing gene expression. They contribute to many physiological processes and pathologies. Consequently, strategies for manipulation of the miRNA pathway are of interest as they could provide tools for experimental or therapeutic interventions. One of such tools could be small chemical compounds identified through high-throughput screening (HTS) with reporter assays. While a number of chemical compounds have been identified in such high-throughput screens, their application potential remains elusive. Here, we report our experience with cell-based HTS of a library of 12,816 chemical compounds to identify miRNA pathway modulators. We used human HeLa and mouse NIH 3T3 cell lines with stably integrated or transiently expressed luciferase reporters repressed by endogenous miR-30 and let-7 miRNAs and identified 163 putative miRNA inhibitors. We report that compounds relieving miRNA-mediated repression via stress induction are infrequent; we have found only two compounds that reproducibly induced stress granules and relieved miRNA-targeted reporter repression. However, we have found that this assay type readily yields non-specific (miRNA-independent) stimulators of luciferase reporter activity. Furthermore, our data provide partial support for previously published miRNA pathway modulators; the most notable intersections were found among anthracyclines, dopamine derivatives, flavones, and stilbenes. Altogether, our results underscore the importance of appropriate negative controls in development of small compound inhibitors of the miRNA pathway. This particularly concerns validation strategies, which would greatly profit from assays that fundamentally differ from the routinely employed miRNA-targeted reporter assays.

## Introduction

MicroRNAs (miRNAs) are genome-encoded 21–23 nucleotides long RNAs. They serve as sequence-specific guides in the microRNA pathway, a post-transcriptional mechanism suppressing gene expression (reviewed in Jonas and Izaurralde, 2015). The miRNA pathway has been implicated in countless physiological processes and pathologies (reviewed in Bartel, 2009). Biogenesis of canonical miRNAs is a two-step process (reviewed in Ha and Kim, 2014). First, primary miRNAs (pri-miRNAs), long pol II transcripts carrying local hairpin structures, are processed into short hairpin precursors (pre-miRNAs) by the nuclear Microprocessor complex. Next, pre-miRNAs exported to the cytoplasm are cleaved by RNase III Dicer to produce 21–23 nucleotides long mature miRNAs, which are loaded on Argonaute proteins (reviewed in Dueck and Meister, 2014). Mammals have four AGO proteins (AGO1-4), which associate with miRNAs (Meister et al., 2004). During target recognition, mammalian miRNAs typically base-pair imperfectly with cognate mRNAs; a functional interaction appears to involve little beyond the “seed” region comprising nucleotides 2–8 of the miRNA (Wee et al., 2012). Imperfect miRNA:mRNA base-pairing results in translational repression followed by substantial mRNA degradation and requires additional AGO-interacting proteins, particularly GW182, to form the full effector complex (miRISC, reviewed in Jonas and Izaurralde, 2015). In contrast, if the miRNA:mRNA base-pairing is perfect, AGO2 can mediate specific endonucleolytic cleavage of the target mRNA in the middle of the sequence base-paired with the miRNA (Liu et al., 2004; Meister et al., 2004; Song et al., 2004).

**Figure 1:**
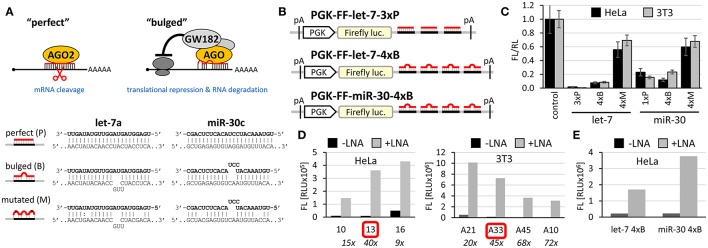
Firefly luciferase reporter systems for HTS. **(A)** “Perfect” and “bulged” binding sites for let-7a and miR-30c miRNAs. A “perfect” site yields perfect base-pairing. If miRNA is bound to AGO2, this type of binding will yield endonucleolytic cleavage in the middle of the base-paired sequence. A “bulged” site is a typical natural miRNA binding site. In this case, silencing of a cognate mRNA requires AGO and associated proteins. Mutated sites carry mutations that should prevent miRNA-mediated repression. **(B)** Schematic structure of let-7 and miR-30 firefly luciferase reporters used for HTS. pA, polyadenylation signal. **(C)** Reporter activities upon transient co-transfection with plasmids expressing firefly luciferase without specific miRNA binding sites. The experiment was performed three times, data were normalized to co-transfected non-targeted *Renilla* luciferase, and the control (firefly luciferase reporter without any miRNA binding sites) was set to one. Except for the control and 1xP miR-30 reporters, which utilized the SV40 promoter and 3′ UTR, all other reporters were driven by the PGK promoter and had BGH 3′ UTR. Error bar = standard deviation (SD). **(D)** Firefly activity test of specific HeLa and 3T3 clones stably expressing PGK-FF-let-7-3xP reporter upon let-7 repression with let-7 LNA inhibitors (50 nM). Clones 13 and A33 were selected for HTS. **(E)** Firefly activity test of specific HeLa cells stably transfected with PGK-FF-let-7-4xB or PGK-FF-miR-30-4xB reporters upon miRNA repression with LNA inhibitors (50 nM). Firefly luciferase activities are displayed as relative luciferase units (RLU) recorded by the luminometer.

Cell-based miRNA reporter systems, which emerged in early miRNA studies (Wightman et al., 1993), typically monitor the RNA silencing activity in cells through a transiently or stably expressed reporter mRNA, which combines a reporter coding sequence (CDS) with a 3′ UTR carrying miRNA binding sites. It has been shown that the number of small RNA binding sites and their types influence the silencing efficiency (e.g., Pillai et al., 2004, 2005). Either of the two above-mentioned types of small RNA:cognate RNA interaction can be used in a reporter (Figure 1A). Accordingly, we will refer as “perfect” to small RNA binding sites that are subjected to endonucleolytic cleavage, for which a perfectly complementary small RNA loaded on AGO2 is sufficient. “Bulged” sites, in contrast, have imperfect base-pairing with small RNAs and mediate translational repression and mRNA degradation, which requires the aforementioned GW182 and other AGO-associated proteins. Such reporters can be repressed either by artificially supplied small RNAs, or by naturally occurring endogenous miRNAs. CDS may encode any protein that can be quantified—by fluorescence, luminescence, or other enzymatic activity.

There are several strategies available for global or specific miRNA modulation (reviewed in Svoboda, 2015). A chemical biology approach would aim at influencing the miRNA pathway with small chemical compounds. Around 100 putative small compound inhibitors and stimulators of the miRNA pathway were reported, so far (reviewed in Deiters, 2010; Di Giorgio et al., 2016).

Identification of putative small compound regulators through high-throughput screening (HTS) requires an efficient, safe, robust and low-cost assay. Several cell-based assays for monitoring miRNA activity *in vitro* have already been developed and yielded potential small compound modulators of RNA silencing. For example, Chiu et al. used dual-fluorescence assay for RNAi induction to screen a chemical library of substituted dihydropteridinones and identified a nontoxic, human cell-permeable, and reversible inhibitor of the RNAi pathway, called ATPA-18 (Chiu et al., 2005). Watashi et al. developed a cell-based assay employing a firefly luciferase mRNA-targeting shRNA transiently transfected into the cells together with firefly reporter and *Renilla* control reporter (Watashi et al., 2010). Upon testing 530 compounds, they identified polylysine and trypaflavine as potential suppressors of miRISC activity (Watashi et al., 2010). Connelly et al. developed and used a miR-122-based *Renilla* luciferase assay to identify miR-122-specific inhibitors; unfortunately, these authors did not reveal how many compounds acted at 10 μM concentration as general miRNA pathway inhibitors (Connelly et al., 2012; Connelly and Deiters, 2014). However, none of the general miRNA inhibitors have received wider recognition and become a common tool.

Here, we present the development and use of high-throughput cell-based firefly luciferase reporter systems for monitoring the activity of endogenous let-7 or miR-30 miRNAs. The reporter systems were tested on 12,816 compounds in human HeLa and mouse NIH 3T3 cells. Let-7 and miR-30 miRNAs were chosen as good candidates for setting up reporters as they are abundant in somatic cells and their biogenesis and activities have been well studied (Pasquinelli et al., 2000; Hutvágner and Zamore, 2002; Zeng et al., 2002, 2005; Zeng and Cullen, 2003, 2004; Pillai et al., 2005). Let-7 is of particular interest because of its tumor suppressor properties and negative impact on pluripotency, which is counteracted in embryonic stem cells and during the establishment of induced pluripotent stem cells (reviewed in Svoboda and Flemr, 2010; Lee et al., 2016). Four primary screens of 12,816 compounds with stably integrated reporters with bulged and perfect miRNA binding sites yielded 248 putative miRNA modulators (82 stimulators and 166 inhibitors), of which we confirmed 163 putative miRNA inhibitors in dose-response assays. However, control assays revealed that the system generates a considerably high proportion of non-specific stimulators of luciferase reporters. We have also found that the stress response, which causes loss of miRNA activity (Bhattacharyya et al., 2006), was rarely caused by the screened compounds. Taken together, we provide a set of luciferase-based assays for future HTS, along with the guidelines for distinguishing false-positive and non-specific luciferase stimulators from the true general miRNA inhibitors.

## Materials and methods

### Cell culture and transfection

Human HeLa cells (ATCC no.: CCL-2), HEK293 (ATCC no.: CRL 1573), and mouse NIH 3T3 cells (ATCC no.: CRL-1658, hereafter named 3T3), were maintained in DMEM (Sigma-Aldrich) supplemented with 10% fetal calf serum (Sigma-Aldrich), penicillin (100 U/mL, Life Technologies), and streptomycin (100 mg/mL, Life Technologies) at 37°C and 5% CO_2_ atmosphere. For delivery of plasmid DNA, cells were plated in a 24-well plate, grown to 40% density and transfected using TurboFect *in vitro* Transfection Reagent (Thermo Scientific) according to the manufacturer's protocol. The ratio of DNA (μg) to TurboFect transfection reagent (μl) was 1:2 for HeLa cells and 1:1 for 3T3 cells. For transfections of miRCURY locked nucleic acid (LNA) miRNA family inhibitors (Exiqon), cells were plated in a 24-well plate, grown to 80% density and transfected using Lipofectamine® 2000 Transfection Reagent (Life Technologies) according to the manufacturer's protocol. Unless stated otherwise, transfected cells were harvested 48 h post-transfection. All firefly luciferase reporters used in HeLa and NIH 3T3 cells are listed in the Table 1.

**Table 1:** Firefly luciferase reporters used in HeLa and NIH 3T3 cells.

**Cells**	**Reporter name**	**Promoter**	**miRNA sites**	**3′ UTR**	**Type**
HeLa	let-7_3xP	PGK	1x let-7B, 2xlet-7P	BGH_polyA	Stable clone
	let-7_4xB	PGK	let-7_4xB	BGH_polyA	Stable pool
	miR-30_1xP	SV40	miR-30_1xP	SV40_polyA	Transient transfection
	miR-30_4xB	PGK	miR-30_7xB	BGH_polyA	Stable pool
	miR-30_4xM	PGK	miR-30_4xM	BGH_polyA	Transient transfection
NIH 3T3	let-7_3xP	PGK	1x let-7B, 2xlet-7P	BGH_polyA	Stable clone
	let-7_4xB	PGK	let-7_4xB	BGH_polyA	Transient transfection
	let-7_4xM	PGK	let-7_4xM	BGH_polyA	Transient transfection
	miR-30_4xB	PGK	miR-30_7xB	BGH_polyA	Transient transfection
	miR-30_1xP	SV40	miR-30_1xP	SV40_polyA	Transient transfection
	Luciferase	SV40	No	SV40_polyA	Transient transfection

### Dual luciferase assay

Luciferase reporter activity was measured with the Dual-Luciferase Reporter Assay (Promega) according to the manufacturer's protocol. Cultured cells were washed with phosphate-buffered saline (PBS) and lysed with the Passive Lysis Buffer (Promega). For 24-well plates, 150 μl of Passive Lysis Buffer was used per well. Modulus Microplate Multimode Reader (Turner Biosystems) was used to measure the luminescence intensity. Luminometric data were adjusted either to the *Renilla* luciferase activity (if the second luciferase reporter was present) or to the total protein amount in lysates measured by the Bradford Protein assay (Bio-Rad) according to manufacturer's instructions. Final data were normalized to a control transfection sample indicated in each experimental result.

### Reporter plasmid construction

#### EGFP reporters

First, forward (MCS_Insert_Fwd) and reverse (MCS_Insert_Rev) oligonucleotides carrying a multiple cloning site were annealed and subcloned into BglII and AgeI sites of the pEGSH.puro plasmid. Next, let-7 1xP forward (1xlet-7P_Fwd) and let-7 1xP reverse (1xlet-7P_Rev) oligonucleotides representing a perfect let-7a binding site were annealed and subcloned into a BglII site of the pCX-EGFP plasmid (Okabe et al., 1997), yielding pCX-EGFP-1xlet-7_1xP; the proper orientation of the let-7a binding site was determined by sequencing. Finally, the EGFP expression cassette was cut out by SalI and HindIII and transferred into the multiple cloning site inserted into the pEGSH.puro plasmid, yielding the final plasmid named pCagEGFP.puro_let-7_1xP. EGFP-based reporter plasmids carrying let-7 2xP, let-7 3xP, and let-7 4xB binding sites were cloned using a similar strategy. Oligonucleotides used for cloning are listed in Table S1.

#### Firefly luciferase reporters

The luciferase reporter plasmids PGK-FL-let-7-3xP-BGHpA, PGK-FL-let-7-4xB-BGHpA, and PGK-FL-miR-30-4xB-BGHpA used to produce reporter cell lines for HTS were built stepwise on the HindIII-AflII pEGFP-N2 (Clontech) backbone fragment using PCR-amplified fragments carrying appropriate restriction sites at their termini. First, we produced a basic firefly luciferase reporter composed of PCR-amplified fragments. The PGK promoter sequence was amplified from the phRL_PGK plasmid (Ma et al., 2010), the firefly luciferase coding sequence was obtained from the pGL4.10 plasmid (Promega), the BGHpA sequence from the pcDNA3.1(–) plasmid (Invitrogen), and a synthetic polyA signal (SpA) sequence upstream of the PGK promoter was taken from the pGL4.10 plasmid. PCR primers used in the cloning are listed in Table S1. Finally, the miRNA binding sites were inserted into the plasmid using *in vitro* synthesized oligonucleotides carrying miRNA binding sites for let-7 or miR-30 miRNA, which were annealed and cloned into a BamHI site downstream of the luciferase CDS; the plasmids were validated by sequencing. The pGL4_SV40_1xmiR-30P plasmid was generated by inserting the fragment with the miR-30 1xP binding site from phRL_SV40_1xmiR-30P (Ma et al., 2010) into pGL4_SV40 using XbaI and ApoI restriction sites.

### Stable reporter cell lines

Stable reporter cell lines had integrated luciferase reporter plasmids carrying a neomycin resistance marker. HeLa or 3T3 cells were plated in a 6-well plate, grown to 40% density and transfected using TurboFect. Transfected cells were cultured for 48 h and stable clones were selected during an additional 2 weeks using 800 μg/ml of G418 in culture media. Upon selection, G418-resistant cells formed a polyclonal population (hereafter referred to as the pool) stably expressing the reporter, which was aliquoted and stored in liquid nitrogen. In the case of PGK-FL-3xlet-7P-BGHpA reporters in HeLa and 3T3 stable cells, clones were isolated, and individual clones with the optimal response were also aliquoted and stored in liquid nitrogen. For HTS and for dose-response experiments, freshly thawed aliquots were cultured and used for experiments without unnecessary passaging.

### Chemical libraries

The primary screening was carried out with 12,816 compounds consisting of two libraries: (I) Bioactive set, collection of 2,816 repurposable compounds with well-characterized biological activities. It consists of the FDA-approved drugs and validated biological probes for various cellular processes (Sigma Lopac 1280, NIH Clinical Collection and Prestwick Chemical Library). (II) Diversity set, collection of 10,000 diverse structures with drug-like properties (Chembridge). The compounds were dissolved in DMSO to a final concentration of 1 mM and the library was reformatted to 384-well polypropylene plates (Corning) containing 10 μl of the compound solution in each well.

### Primary high-throughput screening of chemical libraries

The primary screening was carried out in a fully automated robotic platform Cell::Explorer (Perkin Elmer) in a 384-well format. On the day of the experiment, reporter cells were harvested and resuspended into phenol red-free DMEM culture media to obtain a concentration of 200 cells/μl. Twenty-five microliters aliquots of the cell suspension were dispensed to white polystyrene 384-well plates (Corning) by a Multidrop Combi liquid dispenser (Thermo Scientific). The test compounds were transferred from the 384-well compound plates to 384-well assay plates in Janus Automated Workstation (Perkin Elmer) integrated to Cell::Explorer and equipped with 384 Pin tool (V&P Scientific). The final concentration of the screened compounds was 1 μM. Each plate was shaken for 30 s using a plate shaker Variomag (Thermo Scientific) and then incubated at 37°C and 5% CO_2_ in humidified atmosphere for 48 h.

### Dose-response validation experiments

The dose-response validation was done in an extended set of cell-based luciferase reporter assays. Fresh compound solutions of those active in the primary screens were retrieved from the frozen library and diluted in DMSO to ECHO-qualified 384-well cyclic olefin copolymer plates (Labcyte). On the day of the experiment, reporter cells were harvested and resuspended into phenol red-free DMEM culture media at a concentration of 500 cells/μl. Four-microliters aliquots of the cell suspension were dispensed to white polystyrene 1536-well plates (Corning) by a Multidrop Combi liquid dispenser (Thermo Scientific). The test compounds were transferred from the 384-well compound plates to 1536-well assay plates using contact-free acoustic transfer by ECHO 520 (Labcyte) integrated in the fully automated robotic HTS station Cell::Explorer (Perkin Elmer). The compounds were tested in triplicates at eight different concentrations in the range from 10 μM to 5 nM. Each plate was shaken for 30 s using the plate shaker Variomag (Thermo Scientific) and then incubated at 37°C and 5% CO2 in humidified atmosphere for 24 h.

### Luciferase reporter assay

The luciferase activity was determined using One Glo Luciferase assay system (Promega). Each assay plate was removed from the incubator and was adjusted to room temperature for 10 min. A 15 or 2.5 μl aliquot of ONE-Glo™ Luciferase Assay System reagent for 384- or 1536-well plate respectively, was dispensed to each well using liquid dispenser Multidrop Combi. Assay plates were shaken vigorously for 3 min. After another 10 min, luminescence was recorded on the Envision multimode plate reader (Perkin Elmer). Data were collected and processed with an in-house developed LIMS system ScreenX.

### Cell viability assay

Cell viability was assessed by determining the level of intracellular ATP using the ATPlite™ (Perkin Elmer) luminescent assay. The assay was carried out with 3T3 cells, in parallel with a corresponding reporter assay and under exactly the same conditions as the reporter assay. At the end of the incubation with test compounds, 2.5 μl aliquots of the reagent were dispensed to 1536-well plates using Multidrop Combi. The assay plates were shaken vigorously for 3 min. After another 10 min, luminescence was recorded in the multimode plate reader Envision (Perkin Elmer). Data were collected and processed in the LIMS system ScreenX.

### HTS data analysis

Data were stored on a remote server together with the information about the position of each sample on the plates to enable linking of corresponding compounds with the database of compounds. Data from the primary screen were normalized using the B-score normalization algorithm (Brideau et al., 2003) to remove artifacts arising from the location of the sample on the microtiter plate, such as the edge effect, and/or artifacts arising from the systematic errors introduced by the automated devices used in the HTS process, such as a row and a column effect. The compounds were arranged according to their activity in the HTS experiment, and ~1% of the most active compounds in each experiment, both potential inhibitors and activators of RNA silencing, were selected and tested in the validation dose-response experiments. Data were hierarchically clustered using the Python library *inchlib_clust* and visualized with the corresponding JavaScript library *InCHlib* (Skuta et al., 2014). The clustering was performed with a combination of the Ward's linkage algorithm (Ward, 1963) and the classic Euclidean distance. Cheminformatics software developed by ChemAxon (http://www.chemaxon.com) was used for drawing, displaying and characterizing chemical structures (Marvin Sketch, 17.24, 2017), for structure database management, search and prediction, (Instant JChem, 17.24, 2017), for clustering and diversity analysis of chemical sets (JKlustor, 17.24, 2017), for structure-based property calculation, search and reporting (JChem for Office, 17.23, 2017), and for structure property prediction and calculation (Marvin, 17.24, 2017).

### Stress response analysis

HeLa cells were seeded and transfected in a 6-well dish with RFP-TIA1 (pEmRFP-TIA1 kindly provided by G. Stoecklin) using TurboFect. A day later, ~6,000 transfected HeLa cells per well were seeded in a 348-well plate to get 50–60% confluent cells on the following day when the test compounds were acoustically dispensed to the cells in duplicates by ECHO 520 (Labcyte). The final concentration of compounds was 5 μM. Cells were analyzed at five time points: 0, 2, 6, 12, and 24 h. Cells were first washed with 50 μl of 1x PBS and then fixed with 3.7% PFA (in 1x PBS, pH 7.4) in the dark for 25 min at room temperature (RT). Fixed cells were washed twice with 50 μl of 1x PBS, stained with DAPI in the dark for 5 min at RT, washed once with 1x PBS, and stored in 50 μl of 0.025% sodium azide in 1x PBS. Cell images were acquired using an Olympus Cell^∧^R/Scan^∧^R microscope with 20x/0.45 LUCPLFN PH1 objective. TIA1 localization in the stress granules was analyzed with Scan^∧^R software and validated by personal image inspection and manual analysis.

## Results

### Development of cell-based reporter systems for HTS

To develop reporters for miRNA activity for HTS, we opted for well-established “perfect” and “bulged” binding sites for let-7 and miR-30 miRNAs in previously developed reporters (Pillai et al., 2005; Ma et al., 2010; Figure 1A). Perfect complementarity sequences match let-7a and miR-30c. Some paralogs of the miRNAs would also be able to cleave the cognate sequence, while others that would not have perfect complementarity in the middle of the cognate sequence would recognize the binding site as bulged (Figure S1). Before we decided to use luciferase reporters, we also examined enhanced green fluorescence protein (EGFP)-based reporters. Fluorescence-based reporters would allow for screening living cells and convenient data collection at multiple time points. Accordingly, we tested EGFP reporters varying in promoters and types and numbers of let-7 binding sites (Podolska, 2015); a system with the CAG promoter (Niwa et al., 1991) and three perfect let-7 binding sites was further studied in HEK293 and HeLa cells (Figure S2A). The EGFP-based reporter was sensitive to miRNA pathway repression by AGO2 knock-down, plant virus-derived suppressors of RNA silencing P19 and P21, and miRCURY LNA inhibitors targeting let-7 (Figures S2B–D). We also tested ATPA18, one of the previously reported miRNA inhibitors (Chiu et al., 2005), but it had a minimal if any silencing effect on the reporter at 100 μM concentration. Altogether, while the reporter could sense miRNA-mediated repression, the sample heterogeneity and a relatively small dynamic range of fluorescence readouts in flow cytometry (Figure S2) argued against choosing an EGFP-based system for HTS.

Next, we examined different reporter designs, including individual *Renilla* and firefly luciferase plasmid reporters as well as dual luciferase plasmid reporters expressing both luciferases from one plasmid, where one luciferase would be targeted by miRNA and the other would serve as a negative control (Podolska, 2015). While dual luciferase plasmid designs gave satisfactory results, we opted for a simpler firefly luciferase plasmid-based reporter strategy for HTS (Figure 1B). First, the protocol implementation in our HTS automation system was optimized for measuring a single luciferase reporter in a long-lived luminescence mode. Although this precluded using another luciferase as an internal control, it still provided acceptable reproducibility. Second, a simple luciferase reporter devoid of unnecessary elements that could influence its expression (such as the second luciferase reporter) seemed to be a better choice in this particular case. The choice of the firefly luciferase had another practical consideration—data could be directly compared to the data from firefly luciferase assays from other projects obtained from the same HTS automation system.

Accordingly, we designed firefly luciferase reporters with multiple miRNA binding sites: either three let-7 perfect binding sites or four let-7 or miR-30 bulged sites. Such designs should yield well-repressed reporters that would show a strong increase in expression upon suppression of the miRNA pathway. Indeed, both, let-7 and miR-30 reporters showed good repression relative to non-targeted controls upon transient transfection into HeLa or 3T3 cells (Figure 1C). Next, we produced cells stably expressing the reporters and selected stable cell lines that could be used in HTS. To test the reporter derepression, we employed LNA miRNA family inhibitors targeting let-7 and miR-30 families. Subsequently, for let-7 3x perfect reporters, we chose HeLa and 3T3 clones that showed low raw basal reporter activity, which was strongly stimulated upon transfection of the LNA let-7 inhibitor (Figure 1D). For let-7 and miR-30 bulged reporters, we produced and tested stable HeLa cells but without specific clonal selection (Figure 1E). All three reporters depicted in Figure 1B were stably integrated in HeLa cells; the PGK-FF-let-7-3xP reporter was also stably integrated into 3T3 cells.

### HTS of a compound library

**Figure 2:**
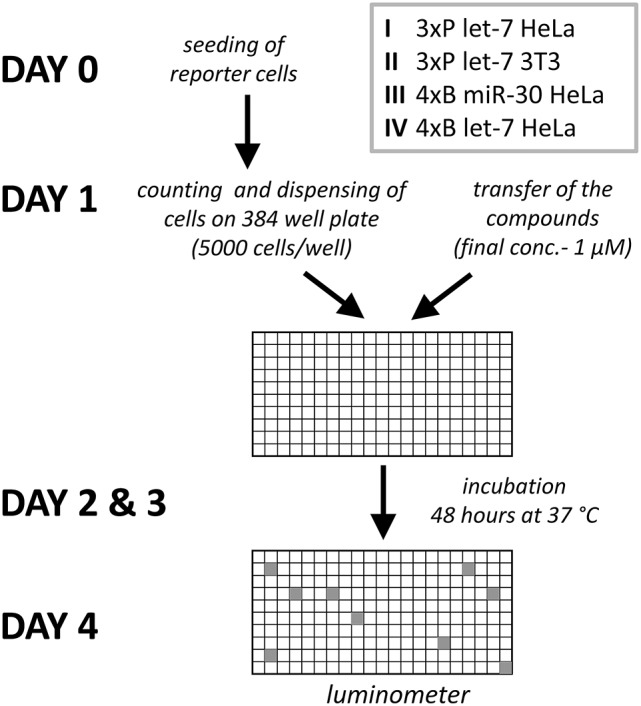
Workflow of HTS with the four miRNA reporter systems.

**Figure 3:**
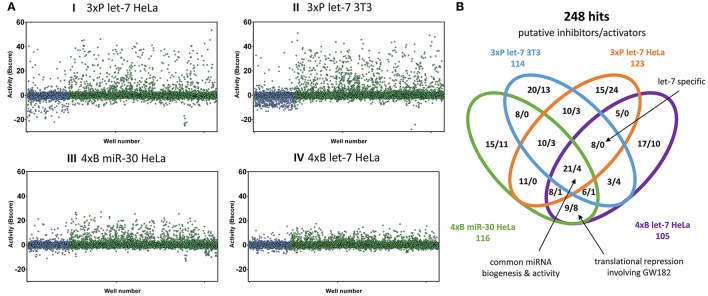
Primary HTS results. **(A)** Overview of all results. Graphs depict normalized reporter activities for all 12,816 compounds. In blue are shown compounds from the bioactive set, while the diversity compound set is shown in green. **(B)** Venn diagram depicting 248 compounds most influencing the indicated reporters. Shown are counts of putative inhibitors (increasing reporter activity) and stimulators (reducing reporter activity).

The four reporter cell lines (I-IV, Figure 2) were produced to be able to distinguish cell type-specific or miRNA-specific compounds and to separate global regulators of the miRNA pathway acting in the miRNA biogenesis or at the level of target repression by endonucleolytic cleavage or translational repression. We then performed four primary HTS experiments (Figure 3A) with a library consisting of a collection of 2,816 well-characterized chemical probes and FDA-approved drugs, and 10,000 structurally diverse, commercially available drug-like small molecules, used at the final concentration of 1 μM.

A striking feature of HTS results was that the bioactive compound collection more frequently reduced the reporter activity, while the diversity set yielded potential miRNA inhibitor candidates at higher numbers (Figure 3A). Reduction of the reporter activity could indicate cytotoxicity or a defect in reporter expression as well as increased miRNA repression. Later validation experiments revealed that there was a considerably higher rate of false positives among the repressors of reporter activity from the bioactive compound library.

Since different assays generated different dynamic ranges of responses, we arranged the active compounds according to their activity in the HTS experiment, and ~1% of the most active compounds, including both activators and inhibitors, were selected for further validation. In total, we identified 248 compounds (166 putative inhibitors and 82 stimulators), which affected the reporter activity in at least one of the four HTS (Figure 3B). Notably, our library contained 18 compounds that were reported to modulate the miRNA pathway previously. However, while 10 of them influenced the reporter activities, only one (6-hydroxy-DL-DOPA) was among the selected 248 compounds (Table 2). These results will be discussed later.

**Table 2:** Reported miRNA pathways modulators present in bioactive and diversity sets.

**Compound**	**Family**	**References**	**Structure**	**Primary HTS activity**	**Positive assay type**	**Primary HTS activity >cut-off**	**Dose response assay**	**Dose response assay type**	**Note**
6-Hydroxy-DL-DOPA	Dopamines	Shum et al., 2012	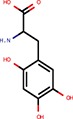	Yes	miR-30-B	Yes	Confirmed	Weak in let-7-B 3T3 only	
Amikacin	Antibiotics	Bose et al., 2013	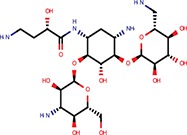	No	Not active	No	–	–	
Apigenin	Flavones	Shibata et al., 2014	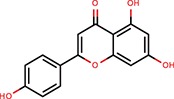	Yes	Bulged reporters	No	–	–	Flavones had many hits
Aurintricarboxylic acid (ATA)		Tan et al., 2012	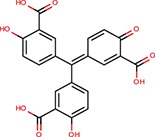	Yes	let-7-P 3T3	No	–	–	
Daunorubicin	Anthracyclines	Maiti et al., 2012	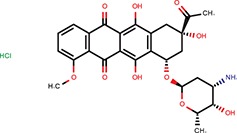	Yes	let-7-P Hela	No	–	–	Idarubicin was a hit
Deoxycorticosterone	Corticoids	Shum et al., 2012	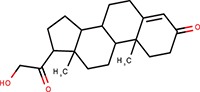	Yes	let-7-P 3T3 activator	No	–	–	Cortisol was a hit
Doxorubicin	Anthracyclines	Maiti et al., 2012	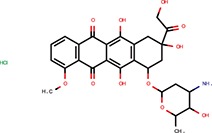	Yes	let-7-P Hela	No	–	–	Idarubicin was a hit
Enoxacin		Shan et al., 2008; Melo et al., 2011	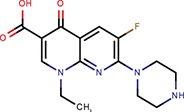	No	Not active	No	–	–	
Epigallocatechin-3-monogallate		Asada et al., 2016	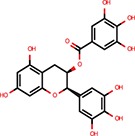	No	Not active	No	–	–	
Ethidium		Maiti et al., 2012	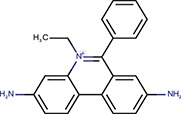	No	Not active	No	–	–	
Flutamide		Shum et al., 2012	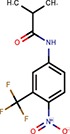	No	Not active	No	–	–	
Kanamycin A	Amtibiotics	Luciferase reporters	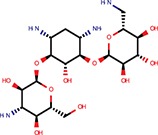	No	Not active	No	–	–	
Neomycin B	Antibiotics	Bose et al., 2013; Tran et al., 2015	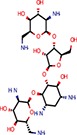	No	Not active	No	–	–	
Pterostilbene	Stilbens	Hagiwara et al., 2012	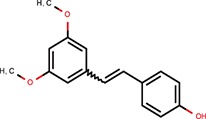	Yes	All reporters	No	–	–	
Rifampicin	Antibiotics	Takahashi et al., 2014	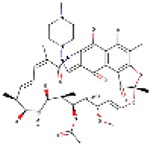	Yes	All reporters	No	–	–	
Streptomycin	Antibiotics	Bose et al., 2012, 2013	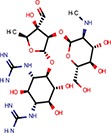	No	Not active	No	–	–	
Tobramycin	Antibiotics	Bose et al., 2013	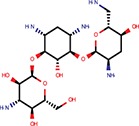	Yes	let-7-P 3T3 activator	No	–	–	
Trans-resveratrol	Stilbens	Hagiwara et al., 2012	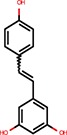	Yes	let-7-B	No	–	–	

### Dose-response analysis of 248 hits from primary HTS

**Figure 4:**
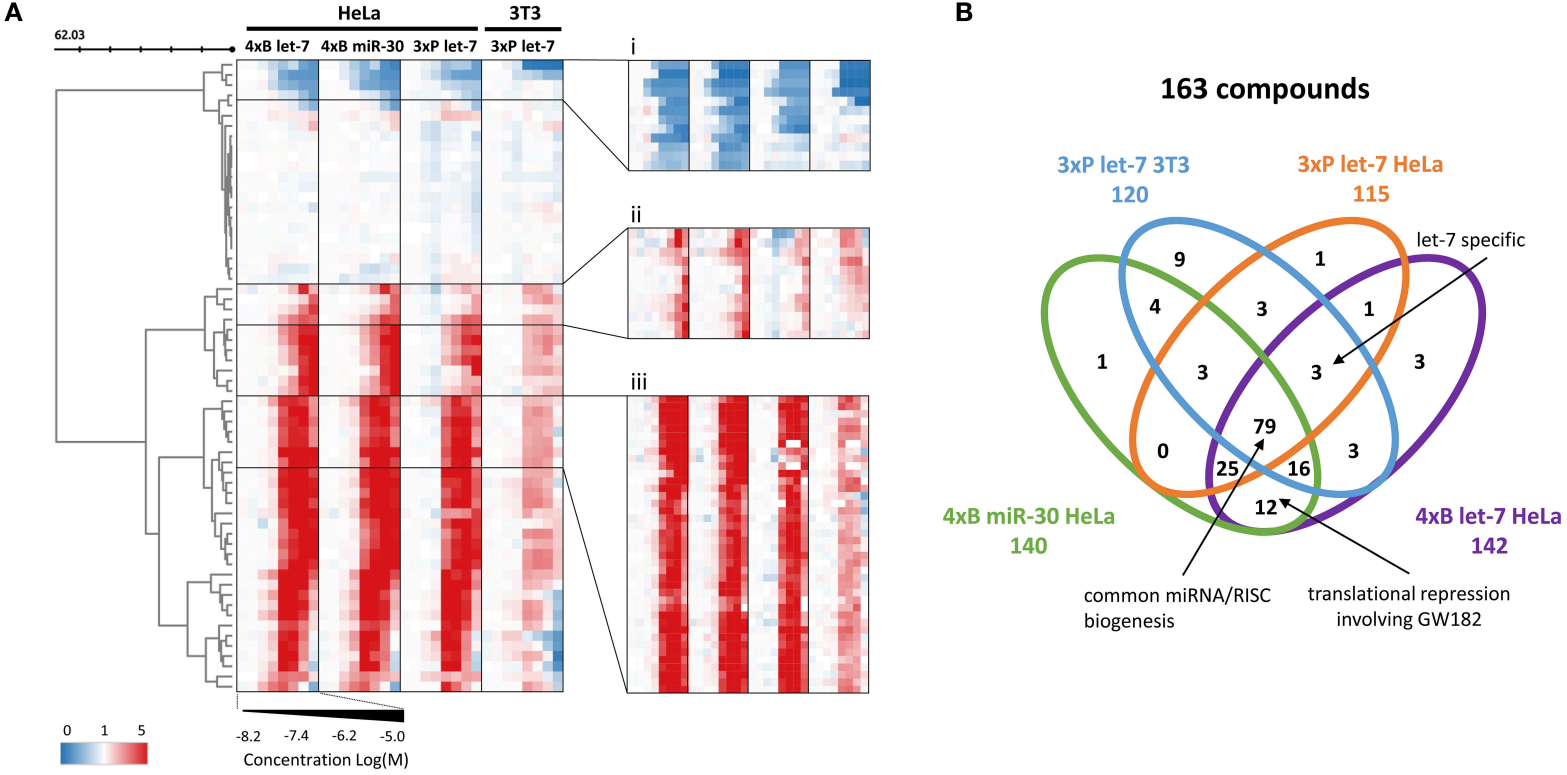
Validation dose-response analysis. **(A)** Cluster analysis of 248 compounds (primary screening hits) based on their concentration-dependent effect on the reporters. The compounds' profiles were compressed according to their Euclidean distance (50 rows) to reveal the main motifs contained in the data. Zoomed clusters (i-iii) depict individual compounds, showing three different concentration-dependent types of effects on the reporters: (i) putative miRNA pathway-stimulating compounds, (ii) less potent putative miRNA pathway inhibitors, (iii) the most abundant activity profile of the putative miRNA pathway inhibitors. **(B)** Venn diagram depicting distribution of the activities of putative miRNA pathway inhibitors in different reporter assays.

Next, we tested the reproducibility of activities of the 248 compounds detected in the primary HTS and repeated the four assays at eight different concentrations from 5 to 10 μM to confirm the results of HTS and examine the potency and specificity of all hits (Figure 4A). The dose-response analysis excluded approximately a quarter of the compounds as false positives. Analysis of the 64 compounds whose activity was not confirmed showed that most of the compounds were from putative miRNA activators and their activities were observed just in a single HTS (Figure S3), indicating that these are likely false positives stemming from stochastic fluctuations of the reporter activities. In total, three quarters (61/82) of the putative miRNA pathway activators were not confirmed. In contrast, most of the putative inhibitors passed the dose-response test; only three compounds were not confirmed. As the screening assay was primarily intended for identification of miRNA pathway inhibitors, we continued further analysis of 163 compounds that showed positive effects in at least one miRNA activity assay (Figure 4B).

Analysis of the confirmed putative miRNA pathway inhibitors showed that compounds that appeared selective for one of the reporter assays often displayed a dose-dependent inhibitory activity in other reporter assays as well. While there were 21 putative inhibitors common for all four HTS experiments (Figure 3B), almost half (79/163) of the compounds showed activity in all four reporter assays, and 120/163 in at least three of them (Figure 4B). In contrast, the above-mentioned 6-hydroxy-DL-DOPA showed some inhibitory dose-response effect only on the 4xB let-7 reporter in 3T3 cells. Apart from these candidates for general miRNA inhibitors, other compounds of interest included let-7-specific inhibitors (3 compounds) and compounds associated specifically with bulged reporters (12 compounds), which could specifically disrupt formation of the full miRISC effector complex. Finally, it should be noted that the dose-response data (Figure 4B) imply that the compound distribution across the earlier Venn diagram (Figure 3B) rather reflects cut-off values than specific compound categories. In other words, the selectivity of the compounds for a specific miRNA family or for a specific mechanism of miRNA inhibition is limited and most compounds are candidates for general inhibitors of miRNA/miRISC biogenesis.

### Minimal impact of the stress response on HTS results

**Figure 5:**
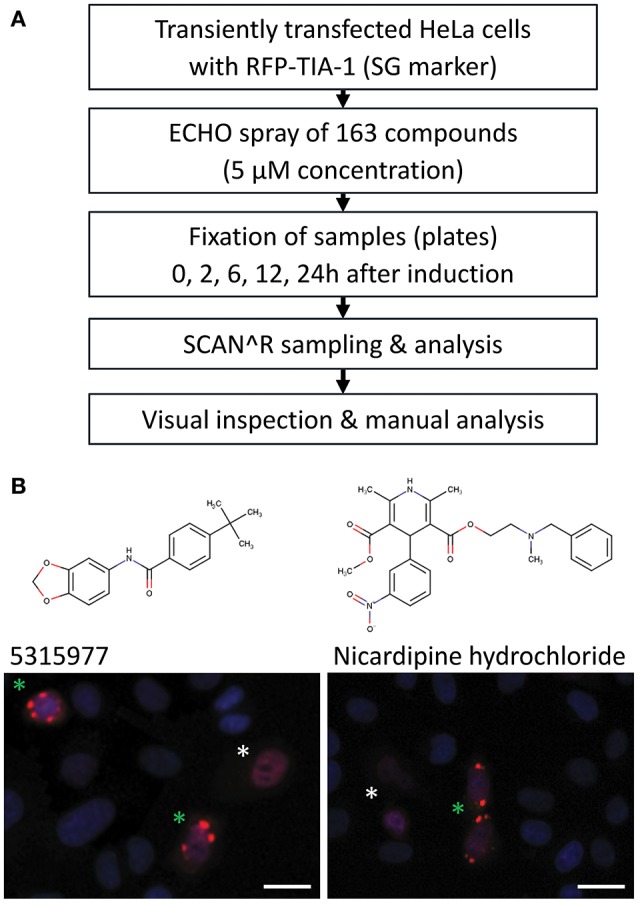
Analysis of induction of stress granules by shortlisted putative miRNA inhibitors in HeLa cells transfected with RFP-TIA-1 reporters. **(A)** Analysis workflow. **(B)** Two compounds that induced >30% stress granule formation in RFP-TIA-positive cells at 5 μM concentration after 6 h. Transfected cells show nuclear localization of TIA-1 (white asterisk), which should change upon induction of stress as TIA-1 relocalizes into stress granules (green asterisk). Scale bars 25 μm.

Indirect inhibition of the miRNA pathway upon stress induction (Bhattacharyya et al., 2006) is a potential mechanism for how compounds could affect the reporter activity in a miRNA-dependent but indirect way. Accordingly, we examined stress induction manifested by formation of cytoplasmic, dynamic mRNA-protein structures called stress granules (SG, reviewed in Protter and Parker, 2016). To screen the compounds for SG formation (Figure 5A), we first transiently transfected HeLa cells with a plasmid expressing red fluorescent protein (RFP)-tagged TIA-1, a stress granule marker that acts as one of SG-nucleating proteins upon stress induction (Kedersha et al., 1999). Next, transfected cells were treated with 5 μM concentration of the 163 putative miRNA inhibitors. The treated cells were fixed at 0, 2, 6, 12, and 24 h time points and analyzed by SCAN^∧^R microscopy for the presence of SGs. Arsenite was used as a positive control for stress induction (Kedersha and Anderson, 2007) (Figure S4). Three independent experiments revealed occasional formation of SGs in ~13% compounds, but only two compounds repeatedly yielded stress granule formation within 6 h in at least 30% of RFP-positive cells (Figure 5B): Nicardipine hydrochloride (Sigma Aldrich) and 5315977 (Chembridge). These data show that stress induction was a rare source of miRNA pathway inhibitors in our HTS assays.

### Reporter response independent of specific miRNA binding sites

**Figure 6:**
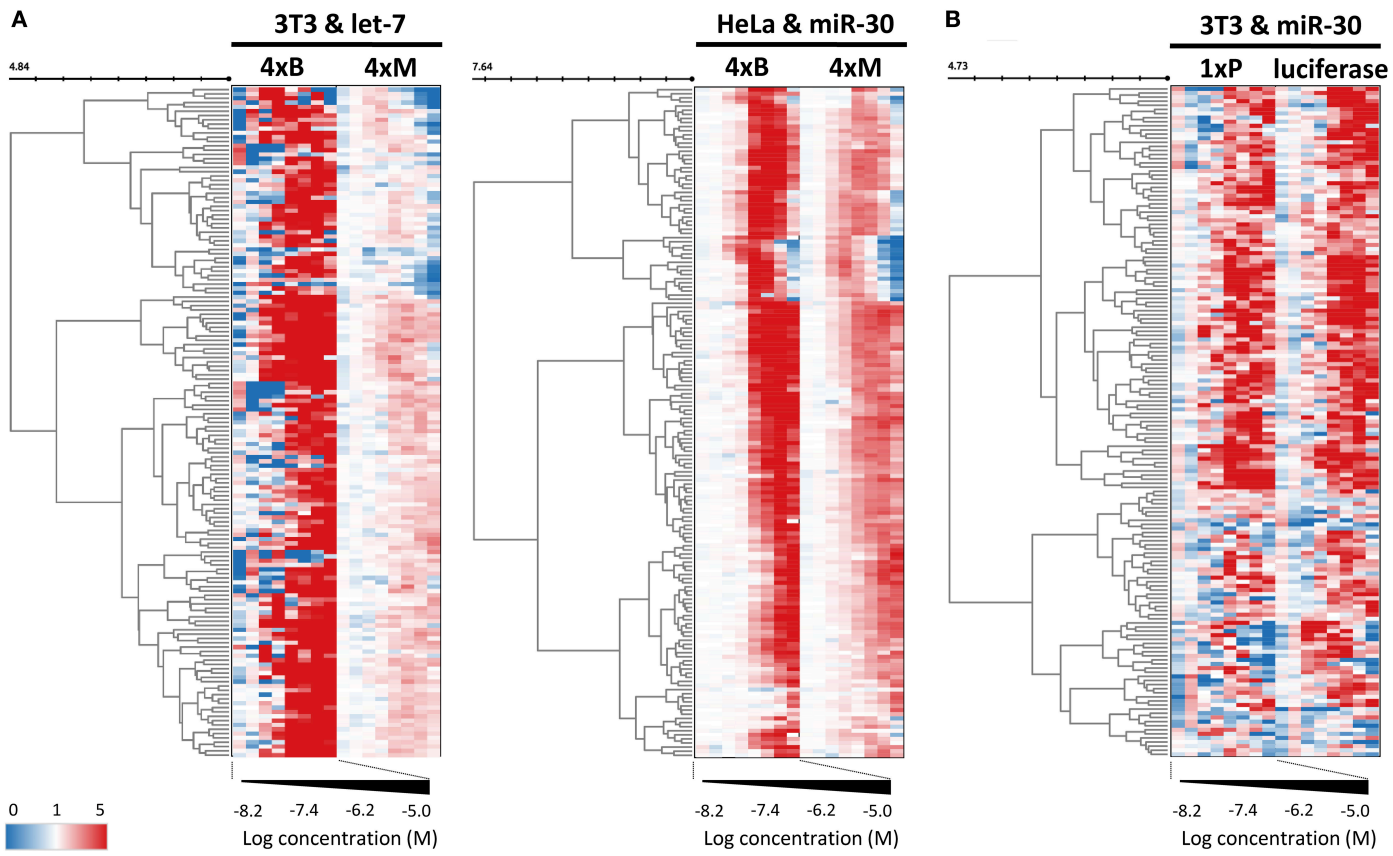
Control reporters reveal high abundance of non-specific (miRNA-independent) hits. **(A)** Heatmap depicting the concentration-dependent response of miRNA-sensitive and insensitive reporter pairs to 163 putative miRNA inhibitors. These reporters utilized the PGK promoter and BGH 3′ UTR. While mutated reporters (4xM) were transiently transfected to HeLa and 3T3 cells, bulged miRNA-sensitive reporters (4xB) were stably integrated. Note that while mutated reporters are generally less upregulated, they yield essentially the same heatmap pattern as the matched reporters with intact miRNA binding sites. The color scale values represent linear relative luciferase activity. **(B)** Heatmap depicting the concentration-dependent response of miRNA-sensitive (1xP) and insensitive (luciferase) reporters that utilized the SV40 promoter and SV40 3′ UTR.

While dose-response experiments confirmed the effects of compounds on the reporter activities, they did not address whether or not reporter responses were directly stemming from the miRNA pathway inhibition. To study the indirect impact of inhibitors on firefly reporters, we thus employed reporter plasmids with mutations in miRNA binding sites that disrupt the miRNA:reporter interaction (Figure 1A). Such “mutated” reporters should be minimally influenced by miRNAs, as evidenced by the apparent relief in expression relative to reporters with intact miRNA binding sites (Figure 1C). First, we performed two dose-response experiments with transiently-transfected mutated reporters. In one experiment, we compared transiently transfected 4xM and 4xB let-7 reporters in 3T3 cells and in another one, we transiently transfected 4xM miR-30 reporter into HeLa cells and compared it with the results of the validation screen where we used HeLa cells stably transfected with 4xB miR-30 reporters (Figure 6A). Importantly, all reporters utilized the PGK promoter and BGH 3′ UTR. Surprisingly, the majority of compounds exhibited a positive dose-response in cells expressing mutated reporters although the magnitude was weaker (Figure 6A). Of the 163 compounds, 69 and 104 showed at least 2-fold increase of the let-7 mutated reporter in HeLa cells and miR-30 mutated reporter in 3T3 cells, respectively. Altogether, 118/163 compounds stimulated at least one mutated reporter at least two-fold. Furthermore, the dose-response trends were highly similar for the majority of the compounds; the pattern was the most striking for the miR-30 experiment in HeLa cells (Figure 6A).

To further examine this observation, we tested firefly luciferase reporters that utilized a different promoter (SV40) as well as a different 3′ UTR sequence (SV40). We used a pair of reporters, one of which had an inserted single miR-30 perfect binding site (1xP miR-30) while the other did not have the insertion (Figure 6B). Importantly, introduction of a single miR-30 site caused at least 80–90% reduction of luciferase activity when compared with the parental reporter plasmid lacking the miRNA binding sites (Figure 1C and Ma et al., 2010). Remarkably, the majority of compounds yielded a comparable impact on luciferase activity regardless of the presence of the miR-30 perfect binding site. Collectively, responses of the reporters that should serve as negative controls revealed an unexpectedly high frequency of promoter and 3′ UTR sequence-independent stimulation of luciferase activity by the tested compounds.

**Figure 7:**
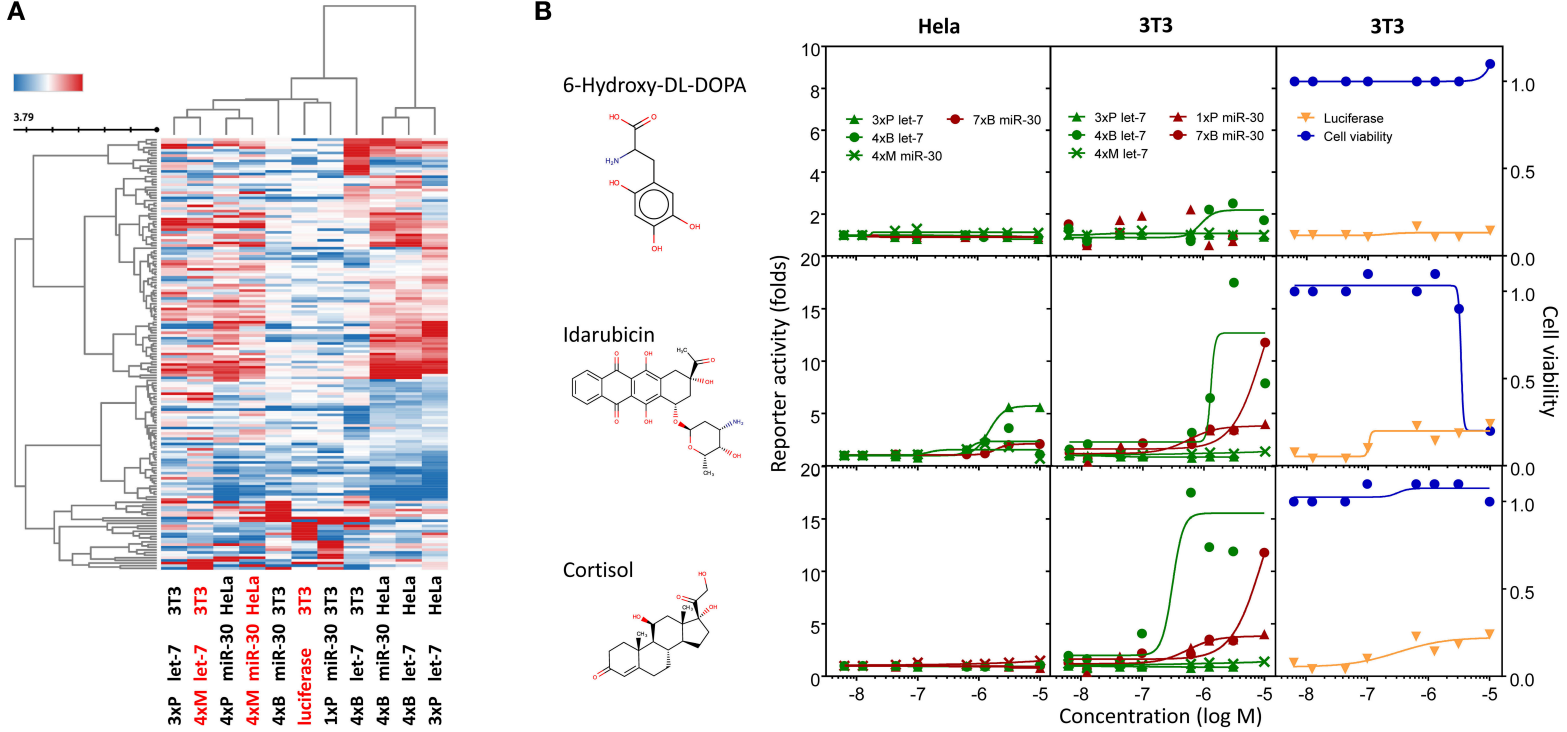
Compounds affecting miRNA reporter assays in a miRNA-independent way are common. Clustering analysis of all dose-response experiments (Table 2) represented by the pIC50 values of 163 compounds. Red font color indicates reporters lacking engineered miRNA binding sites. **(B)** Examples of activities of three compounds related to previously published results. Graphs depict activities of different reporter assays in HeLa and 3T3 cells.

To investigate how miRNA-independent effects impact HTS outcomes, we collected 11 dose-response experiments with the 163 putative miRNA inhibitors and generated hierarchical clustering of the compounds along with individual dose-response sets based on pIC50 values calculated from the dose-response for each compound (Figure 7A). Clustering by luciferase reporter responses in HeLa and 3T3 cells should reveal the major factors affecting the initial selection of 163 putative miRNA inhibitors, such as cell-line choice or reporter whereabouts (transient pool, stable pool, stable clones, bulged, perfect, mutated, or promoter/UTR type).

At the same time, clustering of compounds across all experiments could reveal common reporter stimulators and compounds that would be more likely to affect reporters through inhibition of the miRNA pathway.

The results of the clustering analysis revealed that the contribution of miRNA sensitivity to the general outcome of HTS must be minimal (Figure 7A). Clustering clearly separated stably integrated HeLa reporters from the rest of the samples. This separation was likely influenced by the reporter integration and cell type, but it should be noted that the rest of the samples included two other reporters analyzed in HeLa cells (transiently transfected) and a stably integrated reporter in 3T3 cells. In any case, the most important observation was that mutated reporters clustered together with their miRNA-sensitive variants, i.e., the results obtained with mutated (miRNA-insensitive) reporters were more similar to the results of miRNA-sensitive reporters than to any other mutated reporter (Figure 7A). This is consistent with the analysis of compound clustering, where we did not observe any specific cluster that would exclusively comprise compounds selectively stimulating miRNA-sensitive reporters. This means that (1) the common luciferase reporter-based miRNA assay is prone to high frequency of non-specific hits when it is used for HTS for miRNA inhibitors, and (2) the search for specific hits requires careful analysis of the data as specific hits could be missed when unsupervised compound clustering is employed.

## Discussion

Here we report our experience with HTS for modulators of the miRNA pathway using cell-based luciferase reporter assays. Using a library of 12,816 compounds at 1 μM concentration, we performed HTS experiments in HeLa cells with reporters carrying miR-30 bulged and let-7 bulged and perfect binding sites, as well as an HTS experiment in 3T3 cells with a reporter carrying let-7 perfect binding sites. These primary HTS experiments were followed by 11 dose-response validation experiments with 248 compounds selected from the four primary HTS experiments. Altogether, our assays provide a comprehensive reporter collection for further use in studying small compound modulators of the miRNA pathway.

We chose a common type of luciferase miRNA assay, which has been routinely employed to monitor specific miRNA activity in cells and which had also been adopted for analyzing small molecule inhibitors in the past (Connelly et al., 2012; Connelly and Deiters, 2014). We employed variants of the basic assay, which differed in (1) miRNA (miR-30 and let-7), (2) the type of a binding site (perfect, bulged, mutated, absent), (3) the cell type (human HeLa and mouse 3T3 lines), promoter (PGK and SV40), and 3′ UTR sequence (BHG and SV40). Our results highlight several aspects of this type of reporter system that should be deliberated to optimize the outcome.

In the HTS situation, a miRNA-sensitive luciferase assay essentially explores the space of possible non-specific effects defined by the biological effects of compounds in the tested library. This contrasts with the common use of such an assay for monitoring the miRNA pathway under well-controlled conditions, e.g., when examining the function of a component of the miRNA pathway where it is not expected that miRNA-independent factors would play a significant role. Thus, when a miRNA-sensitive luciferase assay is used in an HTS aiming at miRNA pathway modulators, it has to deal with four categories of hits: false positives, non-specific hits (miRNA-independent), indirect miRNA modulators, and direct miRNA modulators.

False positives would appear during HTS, but would not be reproduced in dose-response validations. False positives in our primary HTS, which was conducted at 1 μM concentration, were low for putative inhibitors (3/166) but high for putative stimulators (61/82). Thus, we can conclude that the rate of false positive hits is minimal when the assay is used for its primary purpose, i.e., in inhibitor screening.

Non-specific hits were represented by compounds reproducibly active in HTS, but in an apparently miRNA-independent fashion. Such compounds would have a similar impact on reporters lacking the miRNA binding sites. In fact, miRNA-sensitive luciferase reporters readily yield such miRNA-independent hits. While it cannot be formally ruled out that mutated reporters themselves could be targeted by other cellular miRNAs, this scenario is unlikely. It would require robust miRNA-mediated repression of reporters with BGH 3′UTR by miRNAs in human HeLa cells and mouse 3T3 cells, as well as repression of the reporters with SV40 3′ UTR, which has an entirely different sequence. Furthermore, the abundance of reporter transcripts from a transiently transfected control luciferase plasmid with PGK or SV40 promoters is likely high (e.g., our previous analysis of transfected SV40-driven luciferase reporters into HEK293 cells (100 ng/well into 24-well plate) showed that luciferase transcripts are 5-10x more abundant than β-actin transcripts (Nejepinska et al., 2012). High luciferase expression levels in control assays are also apparent from the raw luciferase reporter activities in untreated cells across the 11 dose-response datasets, where control assays had two to three orders of magnitude higher readouts than reporters targeted by miRNAs. It is unlikely that control reporters would be efficiently targeted by unknown miRNAs and yet would yield such high expression. In any case, the cause of non-specific luciferase upregulation remains unknown, as it was observed in human and mouse cell lines for two different promoters (PGK and SV40) and two different 3′ UTRs (BGH and SV40). Non-specific inhibitor compounds presumably stimulate some general aspect of reporter expression such as transcription, RNA stability, or translation. Future research should particularly focus on a possible role of vector backbones, which may carry cryptic transcription factor binding sites affecting the reporter transcription (Dougherty and Sanders, 2005; Nejepinska et al., 2012).

While filtering non-specific hits is possible with the assays reported here, they provide only limited means for distinguishing indirect from direct miRNA pathway modulators. Indirect miRNA pathway modulators do not specifically regulate miRNA biogenesis and activity, but some affect signaling or cellular physiology. The miRNA pathway is regulated at different levels (reviewed in Krol et al., 2010), and many regulations are likely yet to be discovered, hence one would expect that many hits would be falling among indirect modulators. Accordingly, our compound assessment included an assay detecting the stress response, a candidate for a common source of indirectly acting miRNA inhibitors (Bhattacharyya et al., 2006). Unexpectedly, stress induction was rare among our candidate miRNA pathway inhibitors. Regarding further differentiation between indirect and direct effects on the miRNA pathway, our reporter assays are limited and need to be complemented with other types of assays analyzing the miRNA biogenesis and activity.

One of the interesting outcomes of our work is the comparison with published data (Table 2). Of 18 compounds reported to affect the miRNA pathway that were present in our compound library, 10 showed activity in HTS, but only one of them (6-hydroxy-DL-DOPA) was picked up for the dose-response analysis. 6-Hydroxy-DL-DOPA was found using an EGFP-based reporter system targeted by perfectly complementary miR-21 (Shum et al., 2012). In our hands, it showed mild inhibitory potential in two of the four dose-response reporter assays in 3T3 cells (1xP miR-30 & 4xB let-7). While it did not show any activity in HeLa cells and two other assays with miRNA-sensitive reporters in 3T3 cells, it also had no effect on mutated reporters (Figure 7B). However, none of the other eight dopamine-related compounds in the library showed any inhibitory activity in HTS assays.

Of the nine previously reported compounds that showed inhibitory activity in HTS but were not picked up for dose-response analysis, two were anthracyclines (doxorubicin and daunorubicin), which were reported to interfere with pre-miRNA processing by Dicer (Maiti et al., 2012). While our earlier HTS for Dicer inhibitors (Podolska et al., 2014) did not identify these compounds (no overlap was found between Bioactives affecting Dicer *in vitro* and luciferase reporters *in vivo*), two other anthracyclines showed inhibitory effects in HTS. One of them (idarubicin) yielded at least three-fold luciferase stimulation in four miRNA-sensitive reporter assays, while it showed a weak stimulation (maximum 2.1-fold) of only one of the three non-targeted reporters (Figure 7B), which makes anthracyclines interesting candidates for further studies.

Deoxycorticosterone was another reported inhibitor (from the same screen as the above-mentioned 6-hydroxy-DL-DOPA; Shum et al., 2012) that showed inhibitory activity in HTS but did not appear in the 1% of the most active compounds picked up for the dose-response analysis. Of interest is that two other corticoids, cortisol and dexamethasone, also acted as inhibitors in HTS, and cortisol was also analyzed in dose-response assays, where it displayed inhibitory activity in 3T3 cells but also seemed to non-specifically stimulate the SV40-driven non-targeted reporter (Figure 7B).

Among the notable matches should also be mentioned stilbenes (pterostilbene and trans-resveratrol), which were reported to be AGO2 stimulators in the past (Hagiwara et al., 2012). This added another potential biological effect to the growing list of resveratrol targets and activities (reviewed in Britton et al., 2015), although the exact molecular mechanism of the miRNA pathway stimulation remains unclear.

While the above-mentioned well-studied compound families that appeared as hits in the literature and our HTS may be worth of further analysis, they are more likely to be indirect regulators of the miRNA pathway than direct and specific ones. At the moment, there is still no commonly used specific miRNA pathway chemical modulator. Our collection of reporter assays provides a cost-effective way for studying effects of chemical compounds while our report provides a framework for filtering the relevant candidate compounds targeting the miRNA pathway.

## Author contributions

KB, DS, JK, KS, and RM: Designed and performed experiments and analyzed data; DS: Designed and performed experiments, Analyzed data, and wrote the manuscript; CS: Analyzed data; PB: Designed experiments and analyzed data; PS: Designed experiments, interpreted data, and wrote the manuscript; all authors contributed to manuscript writing.

### Conflict of interest statement

The authors declare that the research was conducted in the absence of any commercial or financial relationships that could be construed as a potential conflict of interest.
