# Supplementary material for: Cell-Based Reporter System for High-Throughput Screening of MicroRNA Pathway Inhibitors and Its Limitations
Source: Front Genet. 2018 Feb 27;9:45. doi: 10.3389/fgene.2018.00045 (PMC5835079; doi:10.3389/fgene.2018.00045)
Supplement: Supplementary file 1 [file Presentation1.pdf]

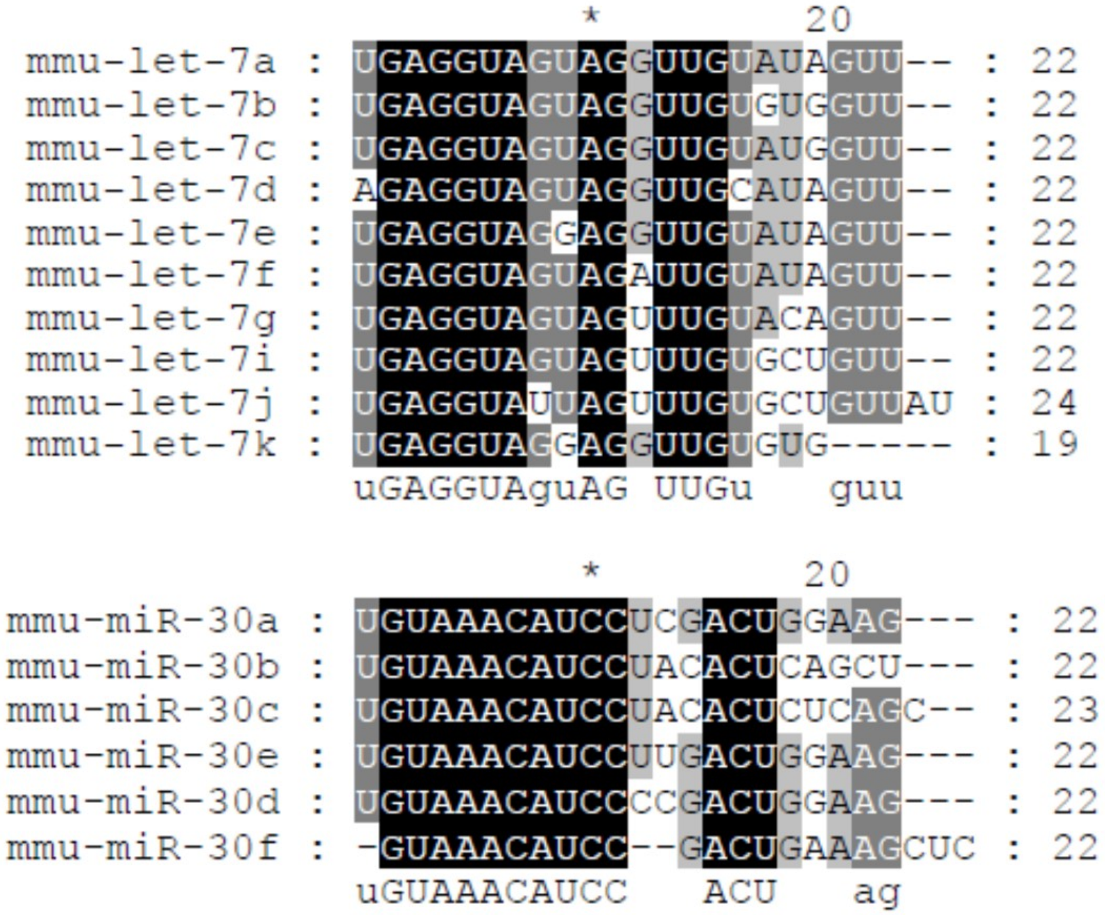

Figure S1 Sequences of let-7 and miR-30 miRNAs. Note that for each “perfect’ binding site, there would be miRNA family members able to cleave it the target as well as other family members, which would engage the target in the typical miRNA fashion.

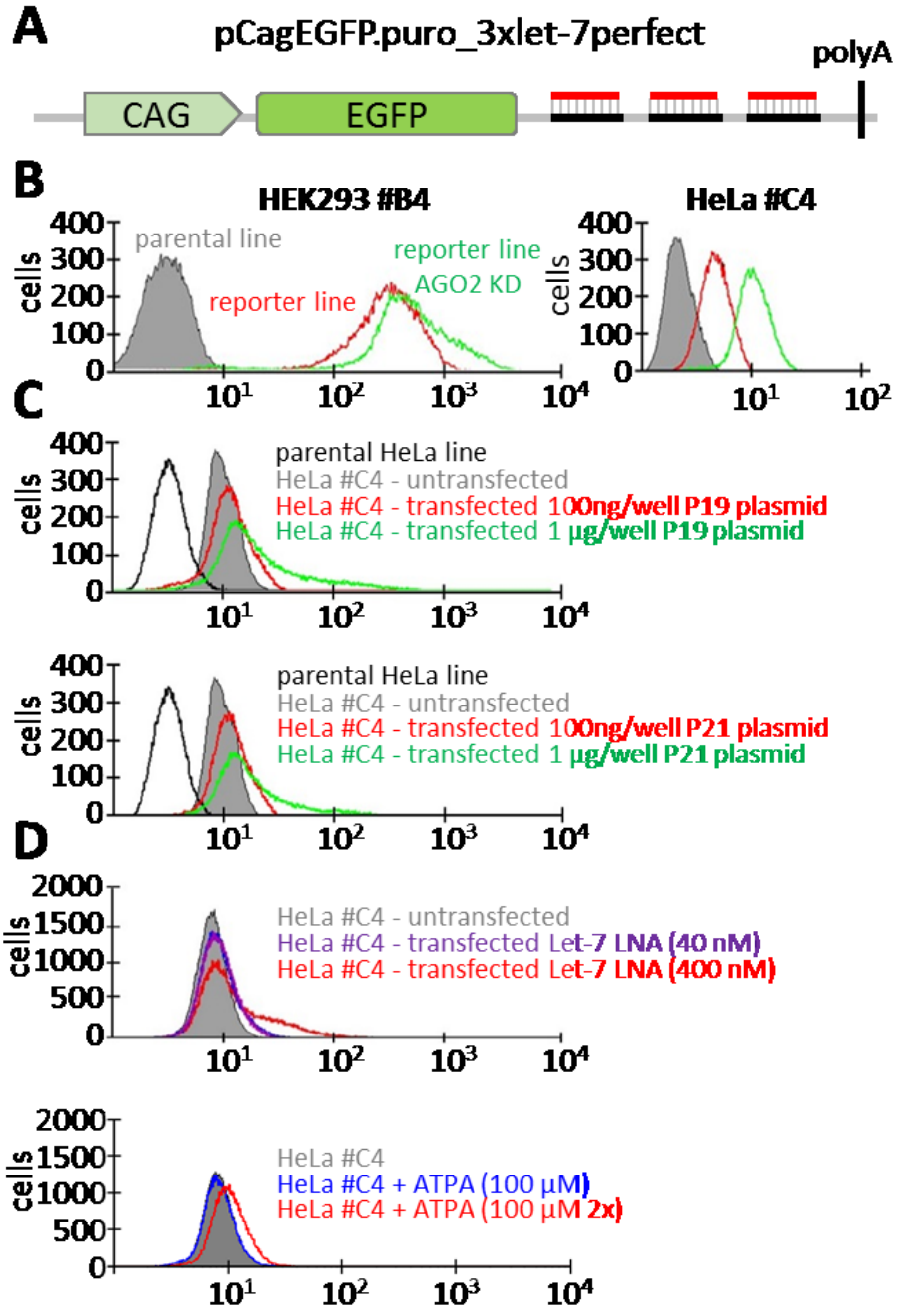

Figure S2 Analyses of EGFP-based reporters. (A) A schematic depiction of a 3xP let-7 EGFP-based reporter. (B) FACS analysis of stable clones in HEK293 cells (#B4) and HeLa cells (#C4). Shown is distribution of EGFP fluorescence signal in untreated reporter lines (red line) and upon AGO2 knock-down using a previously published shRNA expression vector[46]. The grey peak represents signal of the parental cell line lacking EGFP expression. (C) Response of the HeLa #C4 reporter line to expression of plant inhibitors of RNA silencing P21 and P19 (expression vectors were kindly provided by Jozsef Burgyan). (D) Response of the HeLa #C4 reporter line to inhibitory locked nucleic acid (LNA) oligonucleotides suppressing let-7 miRNA family and ATPA18, a previously reported miRNA inhibitor[15]. All experiments were performed in 24-well plates.

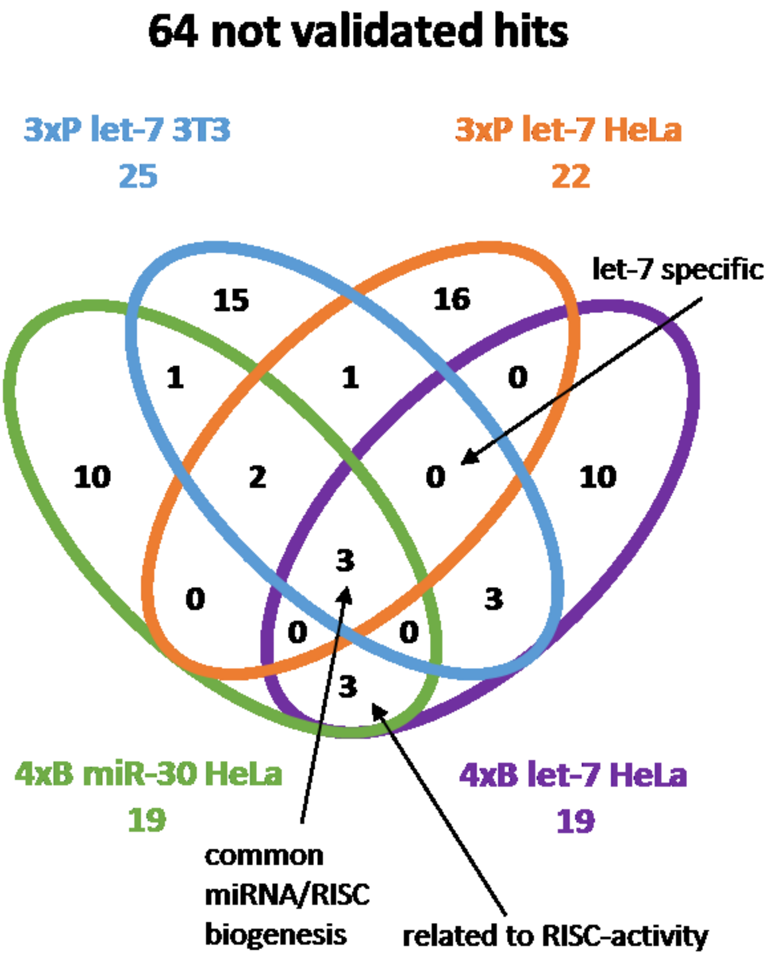

Figure S3 Distribution of false positives among the primary HTS results. The Venn diagram depicts 64 not validated hits, majority of which (51) were identified in only one of the four assays.

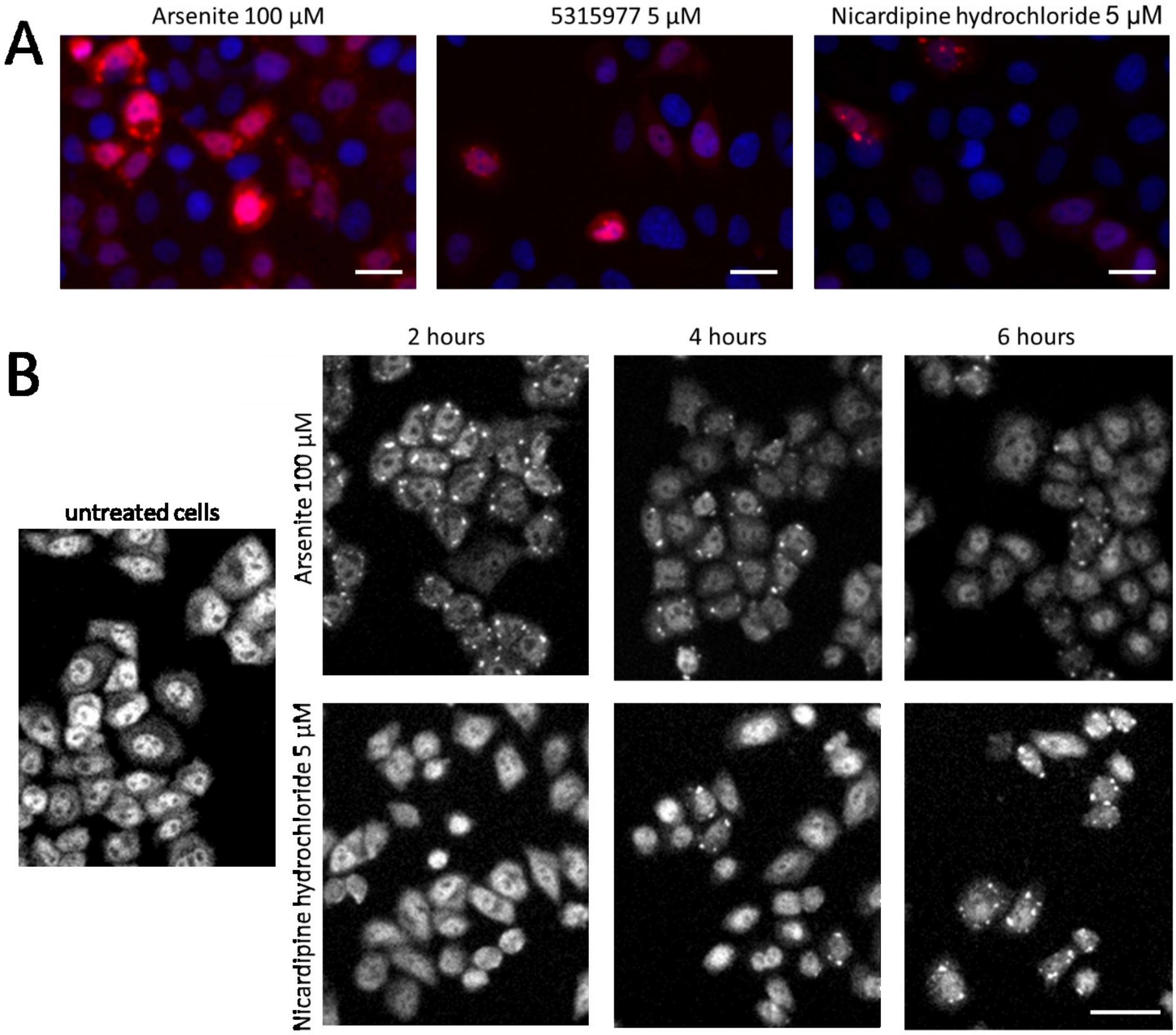

Figure S4 Stress granule induction. (A) Stress granule formation in RFP-TIA-1-positive cells after 2 hours of treatment with arsenite (100 µM), 5315977 (5 µM) and nicardipine hydrochloride (5 µM). (B) Immunofluorescent staining of TIA-1 (sc-1751 antibody from Santa Cruz, diluted 1:200) in non-transfected cells treated with arsenite (100 µM) or nicardipine hydrochloride (5 µM) for 2, 4, and 6 hours. Note different dynamics of stress granule appearance in treatments. All scale bars: 25 µm.

Table S1 Used oligonucleotides

oligonucleotide	sequence (5'->3')	use
MCS_Insert_Fwd	GATCTACATGTCGACTCTTAAGCTTCTCA	EGFP reporters
MCS_Insert_Rev	CCGGTGAGAAGCTTAAGAGTCGACATGTA	EGFP reporters
1xlet-7_Fwd	GATCTACTATACAACCTACTACCTCA	EGFP reporters
1xlet-7_Rev	GATCTGAGGTAGTAGGTTGTATAGTA	EGFP reporters
2xLet-7_Fwd	GATCTACTATACAACCTACTACCTCAATTGCGACTATACAACCTACTACCTCA	EGFP reporters
2xLet-7_Rev	GATCTGAGGTAGTAGGTTGTATAGTCGCAATTGAGGTAGTAGGTTGTATAGTA	EGFP reporters
3xLet-7_Fwd	GATCTACTATACAACCTACTACCTCATCTAGAACTATACAACCTACTACCTCAATTGCGACTATACAACCTACTACCTCAA	EGFP reporters
3xLet-7_Rev	GATCTTGAGGTAGTAGGTTGTATAGTCGCAATTGAGGTAGTAGGTTGTATAGTTCTAGATGAGGTAGTAGGTTGTATAGTA	EGFP reporters
4xlet-7B_Fwd	GATCTACTATACAACCGTTCTACCTCATCTAGAACTATACAACCGTTCTACCTCAATTGCGACTATACAACCGTTCTACCTCACGATTGACTATACAACCGTTCTACCTCA	EGFP reporters
4xlet-7B_Rev	GATCTGAGGTAGAACGGTTGTATAGTCAATCGTGAGGTAGAACGGTTGTATAGTCGCAATTGAGGTAGAACGGTTGTATAGTTCTAGATGAGGTAGAACGGTTGTATAGTA	EGFP reporters
BGH_Fwd_XhoI	GTTCTCGAGTGTGCCTTCTAGTTGCCAGC	luciferase reporters
BGH_Rev_AgeI_AflII	GATCTTAAGACCGGTGCCATAGAGCCCACCGCATC	luciferase reporters
pGL_Fwd_seq	GTGCAAGTGCAGGTGCCAGAAC	luciferase reporters
FL_Rev_NheI-BamHI-SalI	GTAGTCGACGGATCCGCTAGCTCATTACACGGCGATCTTGCCGC	luciferase reporters
PGK_Fwd_AseI	CTCATTAATAGGCGCCAACCGGCTCCGTTC	luciferase reporters
PGK_Rev_HindIII	GTGAAGCTTCCTATAGTGAGTCGTATTAAGTACTTGGGCTGCAGGTCGAAAGG	luciferase reporters
spA_Fwd_AseI_XhoI	GTGATTAATCTCGAGCAATATTATTGAAGCATTTATCAGG	luciferase reporters
spA_Rev_NdeI	GTGCATATGAGAGAAATGTTCTGGCACC	luciferase reporters
3xlet-7P_Fwd	GATCTACTATACAACCTACTACCTCATCTAGAACTATACAACCTACTACCTCAATTGCGACTATACAACCTACTACCTCAA	luciferase reporters
3xlet-7P_Rev	GATCTTGAGGTAGTAGGTTGTATAGTCGCAATTGAGGTAGTAGGTTGTATAGTTCTAGATGAGGTAGTAGGTTGTATAGTA	luciferase reporters
4xlet-7B_Fwd	GATCTACTATACAACCGTTCTACCTCATCTAGAACTATACAACCGTTCTACCTCAATTGCGACTATACAACCGTTCTACCTCACGATTGACTATACAACCGTTCTACCTCA	luciferase reporters
4xlet-7B_Rev	GATCTGAGGTAGAACGGTTGTATAGTCAATCGTGAGGTAGAACGGTTGTATAGTCGCAATTGAGGTAGAACGGTTGTATAGTTCTAGATGAGGTAGAACGGTTGTATAGTA	luciferase reporters
4xmiR-30c_bulge_BglII-Fwd	GATCTGCTGAGAGTGTCAATGTTTACAATTGAGCTGAGAGTGTCAATGTTTACACGATTGCTGAGAGTGTCAATGTTTACATGCATGCTGAGAGTGTCAATGTTTACAA	luciferase reporters
4xmiR-30c_bulge_BglII-Rev	GATCTTGTAAACATTGACACTCTCAGCATGCATGTAAACATTGACACTCTCAGCAATCGTGTAAACATTGACACTCTCAGCTCAATTGTAAACATTGACACTCTCAGCA	luciferase reporters
4xlet-7a-mut_Fwd	GATCTACTGAACAACCGTTCTACGACATCTAGAACTGAACAACCGTTCTACGACAATTGCGACTGAACAACCGTTCTACGACACGATTGACTGAACAACCGTTCTACGACA	luciferase reporters
4xlet-7a-mut_Rev	GATCTGTCGTAGAACGGTTGTTCAGTCAATCGTGTCGTAGAACGGTTGTTCAGTCGCAATTGTCGTAGAACGGTTGTTCAGTTCTAGATGTCGTAGAACGGTTGTTCAGTA	luciferase reporters
4xmiR-30c-mut_fwd	GATCTGCTACGAGTGTCAATGTTACCAATTGAGCTACGAGTGTCAATGTTACCACGATTGCTACGAGTGTCAATGTTACCATGCATGCTACGAGTGTCAATGTTACCA	luciferase reporters
4xmiR-30c-mut_rev	GATCTGGTAACATTGACACTCGTAGCATGCATGGTAACATTGACACTCGTAGCAATCGTGGTAACATTGACACTCGTAGCTCAATTGGTAACATTGACACTCGTAGCA	luciferase reporters
